# Outbreak of 32 *Mycobacterium marinum* infections traced from a single outpatient case: genomic and epidemiological evidence

**DOI:** 10.1080/22221751.2025.2568141

**Published:** 2025-10-14

**Authors:** Jianwei Li, Pengcheng Huai, Fangfang Bao, Zhenzhen Wang, Qing Zhao, Shengli Chen, Xueqing Wang, Yuan Zhang, Jian Liu, Mingjia Hu, Hong Liu, Furen Zhang

**Affiliations:** a Dermatology Hospital of Shandong First Medical University, Jinan City, People’s Republic of China; b Shandong Provincial Institute of Dermatology and Venereology, Shandong Academy of Medical Sciences, Jinan City, People’s Republic of China; c School of Public Health, Shandong First Medical University & Shandong Academy of Medical Sciences, Jinan City, People’s Republic of China

**Keywords:** *Mycobacterium marinum*, outbreak, outpatients, public health, genomic evidence, epidemiological evidence

## Abstract

Outbreaks of *Mycobacterium marinum (M. marinum)* infections associated with recreational water exposure, aquarium maintenance, and fish-related injuries have been increasingly reported, raising concerns as an emerging public health issue in certain regions. The outbreak investigation began with the identification of an index case presenting with papules and nodules on the hand following a sea bass handling injury. Epidemiological tracing focused on seafood exposure, and laboratory confirmation of *M. marinum* infection was achieved through PCR, culture, and histopathology. Whole-genome sequencing was performed on representative isolates to conduct comparative genomic analysis and establish clonal relationships. Between October and December 2020, 32 cases of *M. marinum* infection were identified, after detecting the initial index case. All cases were epidemiologically linked to a single seafood store. Genomic analysis revealed that the outbreak isolates shared >99.5% average nucleotide identity (ANI) with strains from the 2019 Weifang outbreak and exhibited fewer than 20 single-nucleotide polymorphisms (SNPs), confirming their clonal lineage. All patients received appropriate therapy and achieved complete clinical remission within 3–6 months, with no recurrences observed during follow-up (median duration: 6 months). This study underscores the importance of integrating clinical vigilance, epidemiological tracing, and genomic tools to uncover hidden transmission events and guide timely public health interventions. Strengthening hygiene practices, enhancing seafood supply chain monitoring, and promoting protective measures during fish handling are crucial to preventing future outbreak.

*Mycobacterium marinum (M. marinum)* is a well-known pathogenic mycobacterium associated with skin and soft tissue infections [1,2]. It is one of the most common atypical mycobacteria that cause human opportunistic infection [1,2]. The incidence of *M. marinum* infection is steadily increasing worldwide. Outbreaks of *M. marinum* have been reported in the USA, China, and Australia over the past few decades [2]. Here, we report an outbreak of 32 *M. marinum* infections, which was identified following the detection of an initial outpatient case and subsequently confirmed by PCR, culture, and histopathology. Comparative genomic analysis linked this outbreak to the large-scale outbreak in Weifang in 2019 [3].

In October 2020, a patient presented to our hospital with papules and nodules on the hand, accompanied by a history of being stabbed while handling sea bass. Given the patient's history of trauma, characteristic lesion locations, and the fish puncture wounds, infectious diseases, including mycobacterial infections such as *M. marinum*, were prioritized in the differential diagnosis. Notably, the patient reported that several co-workers had developed similar symptoms, raising suspicion of an outbreak. In response, we promptly established an investigation team and initiated epidemiological and diagnostic evaluation. Ultimately, 32 cases of *M. marinum* infection were confirmed by molecular testing (polymerase chain reaction, PCR), mycobacterial culture, and pathological examination (see Supplementary Materials: Table S1).

The study population comprised 10 males (31%), three of whom were employed at a seafood store, and 22 females (69%). All infected individuals developed papules, nodules, or ulcers following injuries caused by sea bass. Fourteen cases (44%) presented with unilateral hand lesions, 18 cases (56%) exhibited bilateral involvement. According to the diagnostic criteria (see Supplementary Materials), 32 cases (100%) were clinically diagnosed cases and 30 cases (94%) were laboratory-confirmed cases. Among the 32 cases, 23 (72%) tested positive via qPCR, and 19 (60%) tested positive by culture, with 12 cases showing positive results for both methods. Histopathological examination of the 32 patients revealed infectious granulomas, with acid-fast bacilli identified in 8 cases (25%). Further epidemiological investigation revealed that all implicated sea bass were traced back to a local aquatic products store in Xiawa Town, Zhanhua District. Strikingly, this same supplier had been linked to a large *M. marinum* outbreak affecting 217 individuals in December 2019 (Figure 1). Genomic analysis confirmed a clonal relationship: isolates from the 2020 (BZ series) and 2019 (SG series) outbreaks shared ≥99.5% average nucleotide identity (ANI). Phylogenetic analysis based on single-nucleotide polymorphisms (SNPs) demonstrated minimal genetic divergence (<20 SNPs between most isolates), providing strong molecular evidence for a common source (see Supplementary Materials: Figure S1, Figure S2, Figure S3, Table S2, Table S3, Table S4, Table S5). All 32 patients were treated with a combination of clarithromycin and rifampicin; minocycline was added for those showing a suboptimal initial response. All patients achieved clinical remission within 3–6 months of treatment, with a median time to remission of 16 weeks. No serious adverse events or disease recurrences were reported during follow-up (median duration: 6 months).

**Figure 1.**
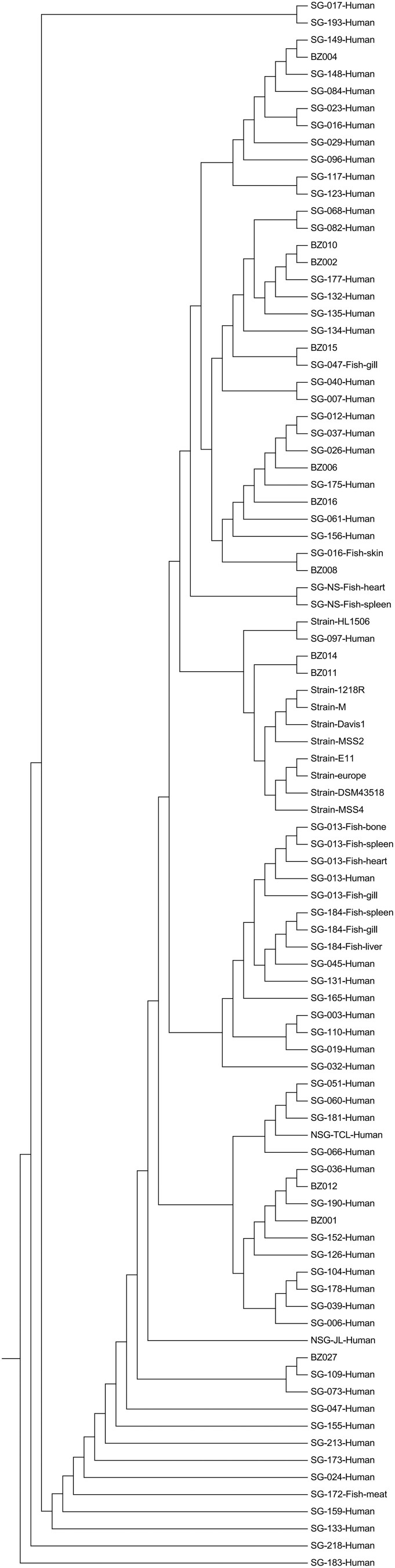
Hierarchical clustering tree of *Mycobacterium marinum* isolates based on pairwise Average Nucleotide Identity (ANI) values. ANI values were calculated using FastANI v1.3, and the resulting similarity matrix was used to construct the tree. The tree includes isolates from the 2019 outbreak (SG series), the 2020 outbreak (BZ series), and reference strains, reflecting their genomic similarity.

Global reports of *M. marinum* outbreaks highlight its emerging role as an environmental pathogen associated with both aquatic exposure and seafood-related injuries. Documented incidents include 217 cases in Weifang (2019) [3], 98 cases in New York City (2013) [4], 18 in Jiangsu Province (2008) [5]. Several nationwide retrospective studies have identified *M. marinum* infections in China(2014–2023) [6], the United States(1996–2014) [7], Denmark (2004–2017) [8], and Satowanese Islanders [9]. Outbreaks have also been reported in laboratory and aquaculture environments involving zebrafish, lungfish, and farmed sturgeon species such as *Acipenser sinensis* and *Acipenser schrenckii* [10–13] (see Table S6). Common seafood vehicles of human infection include sea bass, buffalo fish, carp, and catfish [4]. While infections in Europe and the United States are often linked to aquarium contact or recreational water exposure, domestic cases in China predominantly arise from occupational or household seafood handling. In our study, the outbreak was first identified through clinical suspicion based on a detailed patient history during an outpatient visit. This highlights the importance of thorough clinical evaluation and awareness among frontline healthcare providers. Following the detection of the index case, systematic epidemiological tracing and genomic analysis were performed, confirming the outbreak of 32 cases and establishing a genetic link to the 2019 outbreak. Early detection of index cases can trigger timely public health responses, enabling effective containment.

Following the 2019 outbreak, the implicated seafood store was temporarily closed, and the environment was disinfected. After three months, microbiological testing confirmed that the store met safety standards, allowing seafood sales to resume. However, *M. marinum* is not classified as a notifiable disease under national public health regulations, and as such, there were no routine follow-up inspections or monitoring of the store or its supply chain. This regulatory gap likely contributed to the recurrence of the outbreak in 2020. While this outbreak investigation confirmed the clonal continuity of *M. marinum* across two consecutive years, another limitation is the absence of environmental sampling during the 2020 outbreak. Although the absence of environmental sampling in the seafood supply chain and retail store during the 2020 outbreak may complicate the analysis of the outbreak stages, it does not affect the conclusion that both outbreaks originated from the same batch of fish. With the rising incidence of seafood-associated *M. marinum* infections in China – often linked to traditional fish handling and preparation practices – it is crucial to enhance seafood source monitoring and safety education for both workers and consumers. Strengthening hygiene practices, promoting protective measures during fish handling, and improving surveillance are key to preventing future outbreaks. Specifically, we recommend implementing microbial screening throughout seafood supply chains, particularly for fish that are markedly smaller and thinner than healthy individuals, exhibit khaki-coloured gills in contrast to the bright red gills of healthy fish, display reduced feed intake, lethargy, abnormal swimming behaviour, and exophthalmia. These clinical indicators may indicate potential contamination with *M. marinum*, enabling measures to prevent infection transmission. Furthermore, targeted training for seafood handlers on protective practices, such as glove use and prompt wound care, is essential for reducing further transmission. Taken together, these findings underscore the significance of *M. marinum* as an environmental pathogen and highlight the need for closer integration of clinical, public health, and food safety systems to better address emerging infectious disease threats.

## Ethics statement

The patients in this manuscript have given written informed consent to the publication of their case details.

## Supplementary Material

Supplementary_Materials.doc

## Data Availability

Whole-genome sequencing data supporting this study are available from the corresponding authors upon reasonable request.
