# Supplementary material for: Outbreak of 32 Mycobacterium marinum infections traced from a single outpatient case: genomic and epidemiological evidence
Source: Emerg Microbes Infect. 2025 Oct 14;14(1):2568141. doi: 10.1080/22221751.2025.2568141 (PMC12529748; doi:10.1080/22221751.2025.2568141)
Supplement: Supplementary_Materials.doc [file TEMI_A_2568141_SM9988.doc]

**Supplementary Materials**

[METHODS 2](#__RefHeading___Toc209423379)

[**Diagnostic criteria:** 2](#__RefHeading___Toc209423380)

[**Bacterial Strain and DNA Extraction:** 2](#__RefHeading___Toc209423381)

[**Illumina HiSeq Sequencing:** 2](#__RefHeading___Toc209423382)

[**PacBio Sequencing:** 2](#__RefHeading___Toc209423383)

[**ANI-Based Clustering Analysis:** 2](#__RefHeading___Toc209423384)

[FIGURE 3](#__RefHeading___Toc209423385)

[***Figure S1: Average Nucleotide Identity (ANI) heatmap and cluster tree for Mycobacterium marinum isolates from the 2019 (SG series) and 2020 (BZ series) outbreaks*** 3](#__RefHeading___Toc209423386)

[***Figure S2: SNP-based phylogenetic tree of Mycobacterium marinum isolates from the 2019 (SG series) and 2020 (BZ series) outbreaks*** 5](#__RefHeading___Toc209423387)

[***Figure S3: Geographic distribution of infection cases during the 2020 outbreak in Zhanhua District*** 6](#__RefHeading___Toc209423388)

[TABLE 7](#__RefHeading___Toc209423389)

[***Table S1: Clinical and Laboratory Characteristics of 32 Patients with Mycobacterium marinum Infection*** 7](#__RefHeading___Toc209423390)

[***Table S2: Pairwise SNP difference matrix***
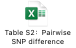
 11](#__RefHeading___Toc209423391)

[***Table S3: PCR detection of Mycobacterium marinum in fish tissues of 2019 outbreak from the implicated supply chain*** 12](#__RefHeading___Toc209423392)

[***Table S4: Primers and probes*** 12](#__RefHeading___Toc209423393)

[***Table S5: PCR Reaction volume*** 12](#__RefHeading___Toc209423394)

[***Table S6: Summary of Reported Mycobacterium marinum Infections in Humans and Animals*** 13](#__RefHeading___Toc209423395)

[REFERENCES 15](#__RefHeading___Toc209423396)

**METHODS**

**Diagnostic criteria:**

1)A history of being punctured by fish or other seafood; 2)Papules,ulcers,or beaded skin nodules along the lymphatic vessels at the site of the puncture wound; 3)Lesional tissue testing positive for *Mycobacterium marinum* via qPCR or culture. Points 1)and 2)correspond to clinically diagnosed cases of *Mycobacterium marinum* infection,while points 1), 2), and 3) represent laboratory-confirmed cases.

**Bacterial Strain and DNA Extraction:**

Mycobacterium marinum strains(e.g.,SG-017-Human, BZ004, SG-013-Fish-gill etc.)were isolated from human clinical specimens or fish tissues(gill,spleen,skin,liver,heart,meat,or bone). Strains were cultured on blood agar plates in a 5%CO<sub>2</sub>atmosphere at 37°C or in fluid medium with agitation. Genomic DNA was extracted from cell pellets using a Bacterial DNA Kit(OMEGA)following manufacturer protocols. Purified DNA underwent quality control(OD<sub>260/280</sub>=1.8–2.0;concentration>6μg)and was quantified with a TBS-380 fluorometer(Turner BioSystems Inc.,Sunnyvale,CA).

**Illumina HiSeq Sequencing:**

For each strain, ≥3 μg genomic DNA was used to construct fragment libraries. DNA was sheared into ~400-bp fragments (Covaris), end-repaired, and ligated to Illumina adapters. Libraries were size-selected via gel electrophoresis, PCR-enriched with index tags, and quality-checked. Paired-end sequencing (PE150) was performed on an Illumina HiSeq platform (Shanghai BIOZERON Co., Ltd).

**PacBio Sequencing:**

For long-read sequencing, 8 μg DNA was fragmented in a Covaris g-TUBE (6,000 RPM, 60 sec). Fragments were end-repaired, ligated to SMRTbell adapters (Pacific Biosciences), and purified with Agencourt AMPure XP beads (Beckman Coulter). Libraries were sequenced on a PacBio RS instrument (Pacific Biosciences, CA).

**ANI-Based Clustering Analysis:**

Average Nucleotide Identity (ANI) was calculated pairwise across all 97 genomes (e.g., human/fish isolates, environmental strains) using JSpecies v1.2.1 with the ANIb (BLAST+) algorithm and default parameters (1020-bp fragment size; ≥70% alignment coverage; ≥30% identity). ANI values were converted to a distance matrix (Distance = 100 − ANI). Hierarchical clustering was performed via the UPGMA algorithm (unweighted pair group method with arithmetic mean) in MEGA X. Dendrogram visualization was generated to delineate genetic relationships between Streptococcus strains from diverse hosts (human/fish) and environments.

**FIGURE**

***Figure S1: Average Nucleotide Identity (ANI) heatmap and cluster tree for Mycobacterium marinum isolates from the 2019 (SG series) and 2020 (BZ series) outbreaks***

The heatmap depicts the genomic similarity among isolates, with values >99.5% indicating a common strain origin. Isolates from the 2019 outbreak (SG series) and the 2020 outbreak (BZ series) are labeled in the figure. The cluster tree illustrates the genetic relationships among the isolates, supporting the conclusion that both outbreaks were caused by the same clonal lineage.


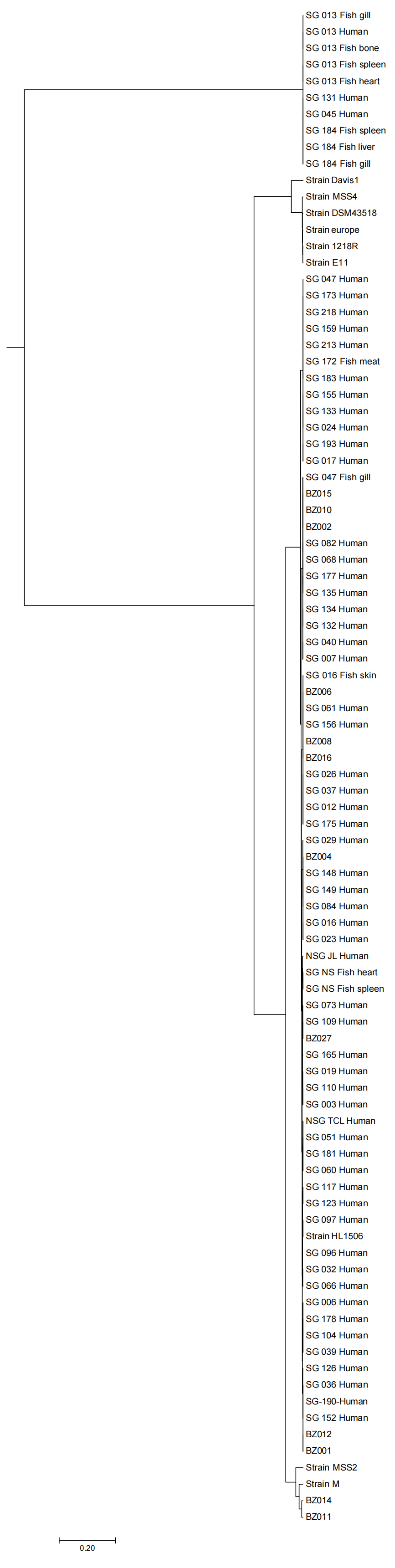


***Figure S2: SNP-based phylogenetic tree of Mycobacterium marinum isolates from the 2019 (SG series) and 2020 (BZ series) outbreaks***

The phylogenetic tree was constructed using core genome SNPs with 1,000 bootstrap replicates to assess the robustness of the inferred relationships. The analysis demonstrated minimal genetic divergence (<20 SNPs) between isolates from the two outbreaks, providing strong molecular evidence for a common strain origin.


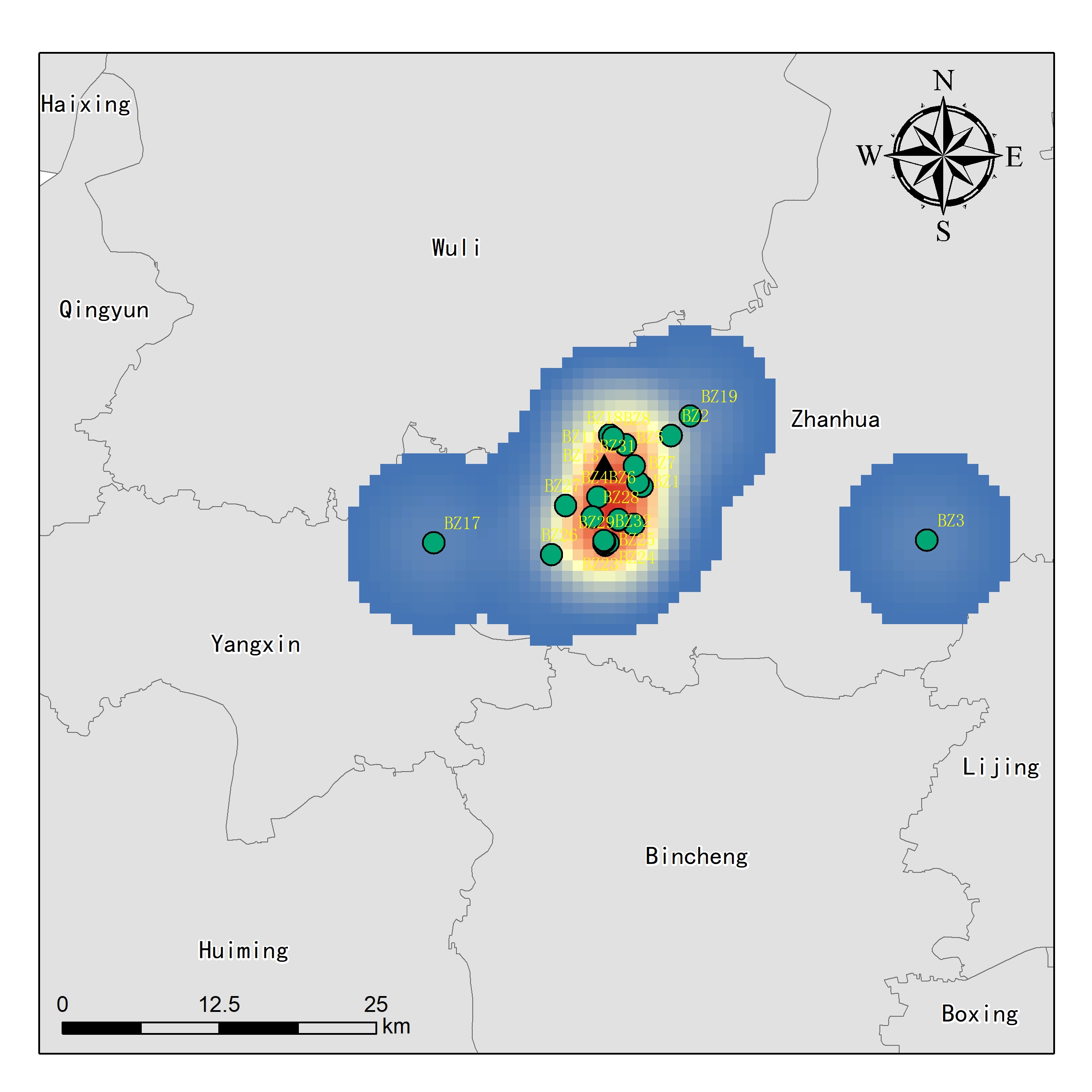


***Figure S3: Geographic distribution of infection cases during the 2020 outbreak in Zhanhua District***

The heatmap depicts the spatial distribution of 32 infection cases across the region, with darker shades representing areas of higher case density. Green markers indicate patient residences, labeled with "BZ+number" codes (e.g., BZ1, BZ2) to identify individual cases. Black triangles denote the locations of seafood stores associated with the outbreak. Notably, the seafood store marked by a black triangle overlaps geographically with clustered patient residences, suggesting a potential spatial association between the infection source and case distribution.

**TABLE**

***Table S1: Clinical and Laboratory Characteristics of 32 Patients with Mycobacterium marinum Infection***

| Patients number | Age | Gender | Incubation period(Days) | Distribution | Clinical presentation | Symptom | Pathological Results | qPCR | *M. marinum* culture | Acid-fast bacilli stain |
| --- | --- | --- | --- | --- | --- | --- | --- | --- | --- | --- |
| BZ001 | 68 | F | 7 | Left hand V finger and Right hand III finger | Solitary papulonodular and granulomatous plaques | Pain | Infectious granuloma, suggestive of NTM | - | + | + |
| BZ002 | 43 | F | 10 | Left hand II finger and Right hand III finger | Solitary papulonodular and ulcerated nodules | Itch | Infectious granuloma | + | + | - |
| BZ003 | 64 | F | 25 | Right hand dorsum | Solitary papulonodular | Pain and Itch | Infectious granuloma | + | + | + |
| BZ004 | 45 | F | 16 | Left hand I finger | Solitary papulonodular | Pain | Infectious granuloma, suggestive of NTM | - | + | - |
| BZ005 | 47 | M | 10 | Right hand dorsum | Solitary papulonodular and granulomatous plaques | None | Infectious granuloma | + | + | - |
| BZ006 | 58 | F | 10 | Left and Right hand dorsum | Solitary papulonodular and granulomatous plaques | Pain | Infectious granuloma | + | + | - |
| BZ007 | 70 | F | 4 | Right hand II,III finger | Solitary papulonodular and ulcerated nodules | None | Infectious granuloma | + | + | - |
| BZ008 | 50 | F | 0 | Left and Right hand dorsum | Solitary papulonodular and granulomatous plaques | Pain | Infectious granuloma, suggestive of NTM | + | + | - |
| BZ009 | 48 | M | 0 | Left and Right hand I finger | Solitary papulonodular | None | Infectious granuloma, suggestive of NTM | + | + | - |
| BZ010 | 69 | M | 2 | Left and Right hand finger | Solitary papulonodular and granulomatous plaques | Pain | Infectious granuloma, suggestive of NTM | - | + | + |
| BZ011 | 94 | M | 0 | Left and Right hand finger | Unknown | Pain | Infectious granuloma | + | - | - |
| BZ012 | 47 | M | 2 | Left hand I finger | Solitary papulonodular | Pain | Infectious granuloma | + | - | - |
| BZ013 | 65 | F | 0 | Left hand dorsum | Solitary papulonodular and ulcerated nodules | Pain | Infectious granuloma, suggestive of NTM | + | + | + |
| BZ014 | 59 | F | 14 | Left hand I finger and forearm | Solitary papulonodular and granulomatous plaques | None | Infectious granuloma, suggestive of NTM | + | + | + |
| BZ015 | 59 | F | 14 | Right hand I finger and forearm | Solitary papulonodular and granulomatous plaques | Pain | Infectious granuloma | + | + | - |
| BZ016 | 56 | F | 10 | Left and Right hand finger, dorsum | Solitary papulonodular and granulomatous plaques | None | Infectious granuloma, suggestive of NTM | + | + | + |
| BZ017 | 51 | F | 13 | Left and Right hand finger,forearm | Solitary papulonodular and granulomatous plaques | Pain | Infectious granuloma | - | + | - |
| BZ018 | 64 | F | 0 | Left hand II,IV finger | Solitary papulonodular | None | Infectious granuloma, suggestive of NTM | + | - | - |
| BZ019 | 40 | F | 0 | Right hand I finger | Solitary papulonodular | Itch | Infectious granuloma, suggestive of NTM | + | - | - |
| BZ020 | 50 | F | 14 | Left and Right hand dorsum,forearm | Solitary papulonodular and granulomatous plaques | Itch | Infectious granuloma, suggestive of NTM | + | - | - |
| BZ021 | 71 | F | 0 | Left hand I finger | Solitary papulonodular | Pain | Infectious granuloma, suggestive of NTM | - | - | + |
| BZ022 | 47 | F | 10 | Left hand V finger and Right hand II finger | Solitary papulonodular and granulomatous plaques | Pain | Infectious granuloma, suggestive of NTM | - | - | + |
| BZ023 | 54 | F | 0 | Right hand II finger | Solitary papulonodular and ulcerated nodules | Pain and Itch | Infectious granuloma | + | - | - |
| BZ024 | 54 | F | 60 | Right hand II,IV finger | Solitary papulonodular and ulcerated nodules | None | Infectious granuloma, suggestive of NTM | + | - | - |
| BZ025 | 30 | F | 20 | Right hand I finger | Solitary papulonodular | Pain | Infectious granuloma | + | + | - |
| BZ026 | 49 | M | 3 | Left hand finger and forearm | Sporotrichoid, focally nodules | None | Infectious granuloma, suggestive of NTM | + | - | - |
| BZ027 | 54 | M | 10 | Left hand I finger and Right hand II finger | Solitary papulonodular | None | Infectious granuloma | + | - | - |
| BZ028 | 54 | M | 6 | Left hand IV,V finger and Right hand II,III,IV finger | Solitary papulonodular and ulcerated nodules | None | Infectious granuloma | + | - | - |
| BZ029 | 63 | M | 10 | Left hand III,V finger and Right II,III finger and forearm | Sporotrichoid, focally and ulcerated nodules | None | Infectious granuloma | - | + | - |
| BZ030 | 62 | F | 10 | Right hand II finger | Solitary papulonodular | None | Infectious granuloma, suggestive of NTM | - | + | - |
| BZ031 | 54 | M | 10 | Left hand and Right hand II finger | Sporotrichoid, focally nodules | None | Infectious granuloma, suggestive of NTM | - | + | - |
| BZ032 | 56 | F | 30 | Left hand III,IV finger and Right hand I finger | Sporotrichoid, focally nodules | Pain | Infectious granuloma | + | - | - |

+ = positive;

- = negative;

F=Female

M=Male

***Table S2: Pairwise SNP difference matrix***

***Table S3: PCR detection of Mycobacterium marinum in fish tissues of 2019 outbreak from the implicated supply chain***

| Fish ID & Status | Tissue Type | qPCR  (Tissue) | qPCR  (Culture) | Sequencing Result(Culture) |
| --- | --- | --- | --- | --- |
| Diseased Fish 1 | Spleen | + | + | Not sequenced |
| Diseased Fish 1 | Skin | + | Not sequenced | Not sequenced |
| Diseased Fish 1 | Heart | + | Not sequenced | Not sequenced |
| Diseased Fish 10 | Heart | - | + | *Mycobacterium marinum* |
| Diseased Fish 17 | Heart | + | + | Not sequenced |
| Diseased Fish 19 | Gill | + | + | Not sequenced |
| Normal Fish 7 | Gill | + | Not sequenced | Not sequenced |
| Normal Fish 8 | Gill | - | Not sequenced | Not sequenced |
| Diseased Fish 9 | Gill | + | Not sequenced | Not sequenced |
| Diseased Fish 11 | Gill | - | Not sequenced | Not sequenced |

+ = positive;

- = negative;

***Table S4: Primers and probes***

| Amplification target | Primer/Probe | Sequence | Base number | Annealing temperature |
| --- | --- | --- | --- | --- |
| mel 2 | Forward-primer | CCGATGCCGATCTTGACTTC | 20 | 60℃ |
|  | Reverse-primer | AGGTCGTGCCAGTCGTTGTC | 20 |  |
|  | Probe | CTTCGGTGGACCGCTGA | 17 |  |

***Table S5: PCR Reaction volume***

| Reaction volume | 1×（μL） |
| --- | --- |
| TaqMan Gene Expression Master Mix | 15 |
| Forward-primer | 0.75 |
| Reverse-primer | 0.75 |
| TaqMan probe | 0.6 |
| ddH2O | 10.9 |
| DNA template | 2 |

***Table S6: Summary of Reported Mycobacterium marinum Infections in Humans and Animals***

| Region | Year(s) | n | Population / Species | Cause of Outbreak /Infection | Discovery of Outbreak /Infection | References |
| --- | --- | --- | --- | --- | --- | --- |
| Case series of humans’ *M. marinum* infection | | | | | | |
| Weifang,China | 2019.12 | 217 | Asian | Cutaneous injury sustained during sea bass handling | Clinical reporting and laboratory confirmation | [1] |
| New York,USA | 2013-2014 | 98 | Western | Handled fish purchased from a Chinese market in New York | Clinical reporting and laboratory confirmation | [2] |
| Jiangsu, China | 2014 | 18 | Asian | Cutaneous injury sustained during contact with sea fish | Clinical reporting and laboratory confirmation | [3] |
| Beijing, China | 2014-2023 | 26 | Asian | Cutaneous injury sustained during sea fish handling | Nationwide retrospective study | [4] |
| North Carolina, USA | 1996-2014 | 28 | Western | Cutaneous injury sustained during aquarium maintenance | Nationwide retrospective study | [5] |
| Denmark | 2004-2017 | 55 | Western | Cutaneous injury sustained during aquarium maintenance | Nationwide retrospective study | [6] |
| Satowan Island, Micronesia | Unknown | 39 | Western | Exposure to suspicious water | Retrospective outbreak investigation | [7] |
| Case series of animals’ *M. marinum* infection | | | | | | |
| London, England | 2013 | 7 | Lungfish | Presumed contaminated water | Laboratory confirmation | [8] |
| Taiwan, China | 2014.11-2015.1 | 14 | Paramesotriton hongkongensis | Presumed contaminated water | Laboratory confirmation | [9] |
| New York, USA | 2010 | Multiple | Zebrafish | Through live feeds, including paramecia, brine shrimp | Laboratory confirmation | [10] |
| Hebei, China | 2009-2010 | 19 | Chinese and Amur sturgeons | Presumed contaminated water | Laboratory confirmation | [11] |

**REFERENCES**

[1] Zhao Q, Bao F, Mi Z, et al. An outbreak of Mycobacterium marinum infection associated with handling seabass in China. Chin Med J (Engl). 2022;135(21):2617-2619.

[2] Yacisin K, Hsieh JL, Weiss D, et al. Outbreak of non-tuberculous mycobacteria skin or soft tissue infections associated with handling fish - New York City, 2013-2014. Epidemiol Infect. 2017;145(11):2269-2279.

[3] Feng Y, Xu H, Wang H, et al. Outbreak of a cutaneous Mycobacterium marinum infection in Jiangsu Haian, China. Diagn Microbiol Infect Dis. 2011;71(3):267-72.

[4] Zhou J, Jia Q, Liu L, et al. Epidemiology and clinical outcomes in skin and soft tissue nontuberculous mycobacteria infections: A retrospective study. J Infect Public Health. 2025;18(3):102655.

[5] Johnson MG, Stout JE. Twenty-eight cases of Mycobacterium marinum infection: retrospective case series and literature review. Infection. 2015;43(6):655-62.

[6] Holden IK, Kehrer M, Andersen AB, et al. Mycobacterium marinum infections in Denmark from 2004 to 2017: A retrospective study of incidence, patient characteristics, treatment regimens and outcome. Sci Rep. 2018;8(1):6738.

[7] Lillis JV, Ansdell VE, Ruben K, et al. Sequelae of World War II: an outbreak of chronic cutaneous nontuberculous mycobacterial infection among Satowanese islanders. Clin Infect Dis. 2009;48(11):1541-6.

[8] Strike TB, Feltrer Y, Flach E, et al. Investigation and management of an outbreak of multispecies mycobacteriosis in Australian lungfish (Neoceratodus fosteri) including the use of triple antibiotic treatment. J Fish Dis. 2017;40(4):557-570.

[9] Li WT, Chang HW, Pang VF, et al. Mycolactone-producing Mycobacterium marinum infection in captive Hong Kong warty newts and pathological evidence of impaired host immune function. Dis Aquat Organ. 2017;123(3):239-249.

[10] Chang CT, Benedict S, Whipps CM. Transmission of Mycobacterium chelonae and Mycobacterium marinum in laboratory zebrafish through live feeds. J Fish Dis. 2019;42(10):1425-1431.

[11] Zhang DF, Ji C, Zhang XJ, et al. Mixed mycobacterial infections in farmed sturgeons. Aquaculture Research. 2015;46(8):1914-1923.
